# Solution-Shearing Self-Assembly of Hydrothermally Synthesized Tellurium Nanowires for Fabricating Field-Effect Transistors with Wafer-Scale Uniformity

**DOI:** 10.3390/nano16191251

**Published:** 2026-10-03

**Authors:** Hao Yang, Mose Park, Yanjin Tang, Hyun-Dong Lee, Ji-Won Kang, Zhiyi Lyu, Hoo-Jeong Lee

**Affiliations:** 1 School of Advanced Materials Science and Engineering, Sungkyunkwan University, Suwon 16419, Republic of Korea; yanghao0393@g.skku.edu; 2 Department of Biophysics, Sungkyunkwan University, Suwon 16419, Republic of Korea; 3 Department of Smart Fab. Technology, Sungkyunkwan University, Suwon 16419, Republic of Korea; pms7879@skku.edu (M.P.); yanjintang@skku.edu (Y.T.); hd5229@skku.edu (H.-D.L.); kangenius@g.skku.edu (J.-W.K.); 4 College of Engineering, Eastern Institute of Technology, Ningbo 315200, China

**Keywords:** one-dimensional tellurium, shearing-guided deposition, directional charge transport, device integration, scalable manufacturing

## Abstract

Tellurium nanowires (TeNWs), a typical p-type semiconductor, have attracted significant attention in field-effect transistors (FETs) due to their narrow bandgap and high carrier mobility properties. However, fabricating large-area electronic devices with uniform performance remains a major challenge. In this study, we report a facile solution-shearing self-assembling strategy for fabricating TeNW-FETs with high-performance and uniformity. The solution-shearing process occurs at the interface during relative motion between the substrate and TeNW dispersion, enabling the TeNWs to reorient and adhere to the substrate, forming a uniform nanowire network. The transfer characteristics of TeNW-FETs reveal highly uniform electrical performance, and FET performance is strongly modulated by dispersion concentrations. Moreover, we investigated the impact of the solution-shearing direction on the device performance. The effective field-effect mobility of FETs with channels perpendicular to the substrate movement direction was approximately 12.1% lower than that of FETs with channels parallel to the movement direction. To further demonstrate scalability, we fabricated transistor arrays containing 324 individual FETs on a 4-inch Si/SiO_2_ wafer, achieving 100% functional device yield and good performance uniformity. The devices exhibited an average I_ON/OFF_ ratio of 6.9 × 10^2^, mobility (μ_eff_) of 24.3 cm^2^ V^−1^ s^−1^, subthreshold swing (SS) of 7.9 V dec^−1^ and threshold voltage (V_TH_) of 12.0 V.

## 1. Introduction

One-dimensional semiconducting nanostructures exhibit tremendous potential for next-generation nanoelectronics owing to their efficient carrier transport properties and mechanical compliance. Recent works have demonstrated notable progress in both *n*-type nanowires (e.g., indium oxide, zinc oxide, niobium oxide) and *p*-type nanowires (e.g., silicon nanowires, carbon nanotubes) for electronic applications [1,2,3,4,5,6,7,8]. Among emerging 1D semiconductors, tellurium has been increasingly recognized as a promising p-type candidate [9,10,11,12]. The characteristics of its high carrier mobility and narrow bandgap (~0.31 eV) have attracted significant attention in the field of electronic and optoelectronic devices [13,14,15]. Tellurium exhibits a trigonal crystalline lattice structure, composed of densely packed atoms arranged in helical chains. These chains are predominantly stabilized by van der Waals interactions. Owing to its distinctive structural characteristics and associated properties, various synthesis methods have been developed to fabricate high-quality tellurium nanostructures, including nanowires, nanosheets, nanotubes, and nanobelts [16,17,18,19].

Among these, one-dimensional tellurium nanowires (TeNWs) can be reproducibly formed through various techniques, including solution-based approaches, vapor-phase growth, and electrochemical deposition. Solution-based approaches include hydrothermal and solvothermal synthesis, self-seeding routes, reflux-based synthesis, hot-injection colloidal synthesis, and microwave-assisted synthesis [20,21,22,23,24,25]. In particular, hydrothermal synthesis utilizes aqueous precursor reduction and growth-directing additives to obtain high-aspect-ratio TeNWs and has been extended to large-scale production [26]. Vapor-phase growth, including physical vapor deposition, chemical vapor deposition, and thermal evaporation/vapor transport, achieves crystalline TeNW growth on substrates, with morphology controlled by sources, temperatures, vapor supply, and growth duration [24,27,28,29]. Electrochemical techniques utilize template-confined nanowire growth or template-free deposition controlled by electrolyte composition, applying potential, and temperature to synthesize TeNWs [30,31,32]. Notably, large-scale tellurium films have been successfully produced and characterized via thermal evaporation [33,34]. However, the functional properties of these tellurium films are highly sensitive to deposition parameters, particularly temperature and rate, which pose significant challenges in achieving highly crystalline films over large, scalable areas. Hence, their large-scale process remains challenging, which limits their applications.

Strategies for fabricating large-scale devices at low temperatures have been reported. For instance, the Yu group [35] utilizes the Langmuir-Blodgett (LB) technique to produce TeNW films over a large-scale area. Through this technique, Ye [36] reported a substrate-free solution process for synthesizing large-area, high-quality 2D tellurium with a TFT mobility of ~700 cm^2^ V^−1^ s^−1^. However, due to the less uniformity and random orientation of the Te layer, the prepared TFT devices cannot achieve uniform electrical performance. Furthermore, Muhammad et al. [37] also developed a low-temperature blade-coating process using a blade-coating to form a nanonetwork on silicon substrates, achieving stable, uniform performance in a 42-device transistor array. Recently, the Zhu group [38] proposed a “locking-and-shearing” strategy, where the silicon substrate surface was modified to generate electrostatic interactions, facilitating the assembly of TeNWs into wafer-scale, aligned arrays. The aligned quality is highly dependent on the immersion rate and dispersion concentration. This strategy requires hydrophilizing the substrate surface and imparting a positive charge to the surface.

Inspired by these shearing strategies, we report a facile solution-shearing strategy for fabricating wafer-scale TeNW-FETs with uniform and good electrical performance. In this study, TeNWs with a diameter of approximately 7.2 nm were synthesized via a hydrothermal method and uniformly dispersed in water. The solution-shearing strategy was then used to extract the Si/SiO_2_ substrate from the TeNW dispersion at a controlled low speed, assembling the TeNWs on the substrate. We also found that the concentration of the TeNW dispersion significantly affects the electrical performance. Furthermore, we fabricated transistor arrays containing 324 FETs on a 4-inch Si/SiO_2_ wafer, the devices exhibiting good electrical performance with an average value of effective field-effect mobility μ_eff_ of 24.3 cm^2^ V^−1^ s^−1^, threshold voltage V_TH_ of 12.0 V, current on/off ratio I_ON/OFF_ of 6.9 × 10^2^ and subthreshold swing SS of 7.9 V dec^−1^. This study demonstrates the large-area fabrication of TeNW-FETs with uniform performance.

## 2. Materials and Methods

### 2.1. TeNW Synthesis

TeNWs were synthesized by a hydrothermal method. Typically, sodium tellurite (Na_2_TeO_3_, 0.1844 g, Sigma-Aldrich, St. Louis, MO, USA) and polyvinylpyrrolidone (PVP) (2.0 g, M_w_ = 40,000, Sigma-Aldrich) were dissolved in 62 mL of deionized water under continuous magnetic stirring at 800 rpm for 15 min, until a homogeneous precursor solution was obtained. Next, hydrazine hydrate (N_2_H_4_·H_2_O, 3.33 mL, 50%, *w*/*w*, Sigma-Aldrich) and aqueous ammonia solution (NH_3_·H_2_O, 6.66 mL, 32%, *w*/*w*, Sigma-Aldrich) were injected and stirred for 15 min. Afterward, the solution was transferred into a 100 mL Teflon-lined stainless-steel autoclave. The autoclave was then sealed and heated at 180 °C for 3 h. After naturally cooling to room temperature, a black TeNW solution was obtained and centrifuged two times: centrifuging at 4000 rpm for 3 min to remove large particles, and further centrifuging at 10,000 rpm for 15 min to collect TeNW precipitates. The collected TeNW precipitates were redispersed into 100 mL of DI water with vigorous fluctuation, and the dispersion was used for further device fabrication. Subsequently, the TeNW precipitates were vacuum-dried at 70 °C for 10 h to obtain TeNW powder for further material characterization.

### 2.2. TeNW-FET Fabrication

The TeNW dispersion was poured into a quartz vessel with geometric dimensions of L × W × H = 11 cm × 1 cm × 10 cm. A p^+^-Si wafer substrate with 200 nm SiO_2_ thickness was cleaned with ethanol, acetone, and isopropanol, followed by N_2_ gas flow drying and UV-zone surface cleaning for 10 min to obtain a highly hydrophilic surface for stable and uniform nanowire assembly. Then, the substrate was quickly immersed in the TeNW dispersion. After fully submerging, the substrate was carefully extracted at a controlled speed of 66.7 μm s^−1^, allowing the TeNWs to self-assemble onto the substrate. Then, the drain and source electrodes were further patterned using a shadow mask, followed by an E-beam evaporation of Cr/Au (5 nm/40 nm). The device was designed with a channel length (L) of 50 μm and channel width (W) of 1000 μm.

### 2.3. Characterization and Electrical Measurement

X-ray diffraction (XRD) was used with Cu Kα radiation (Rigaku, Tokyo, Japan) to confirm the crystalline structure. The morphology of TeNWs were characterized by scanning electron microscopy (SEM, Quanta FEG 200, FEI Company, Hillsboro, OR, USA) and transmission electron microscopy (TEM, JEOL, JEM-2100F, Tokyo, Japan). The chemical states of the TeNWs were analyzed by X-ray photoelectron spectroscopy (XPS) using a Thermo ESCALAB 250 HRXPS instrument (Thermo Fisher Scientific, Waltham, MA, USA). Raman spectra were measured using an inVia confocal Raman microscope (Renishaw, Wotton-under-Edge, UK) with a 532-nm laser excitation source. UV-vis absorption spectra were used to confirm the concentration of TeNW dispersions with a Cary 5000 spectrometer (Agilent Technologies, Santa Clara, CA, USA). Electrical characteristics were measured using a Keithley 4200B-SCS parameter analyzer under ambient conditions at room temperature (Keithley Instruments, Cleveland, OH, USA).

## 3. Results

**Figure 1:**
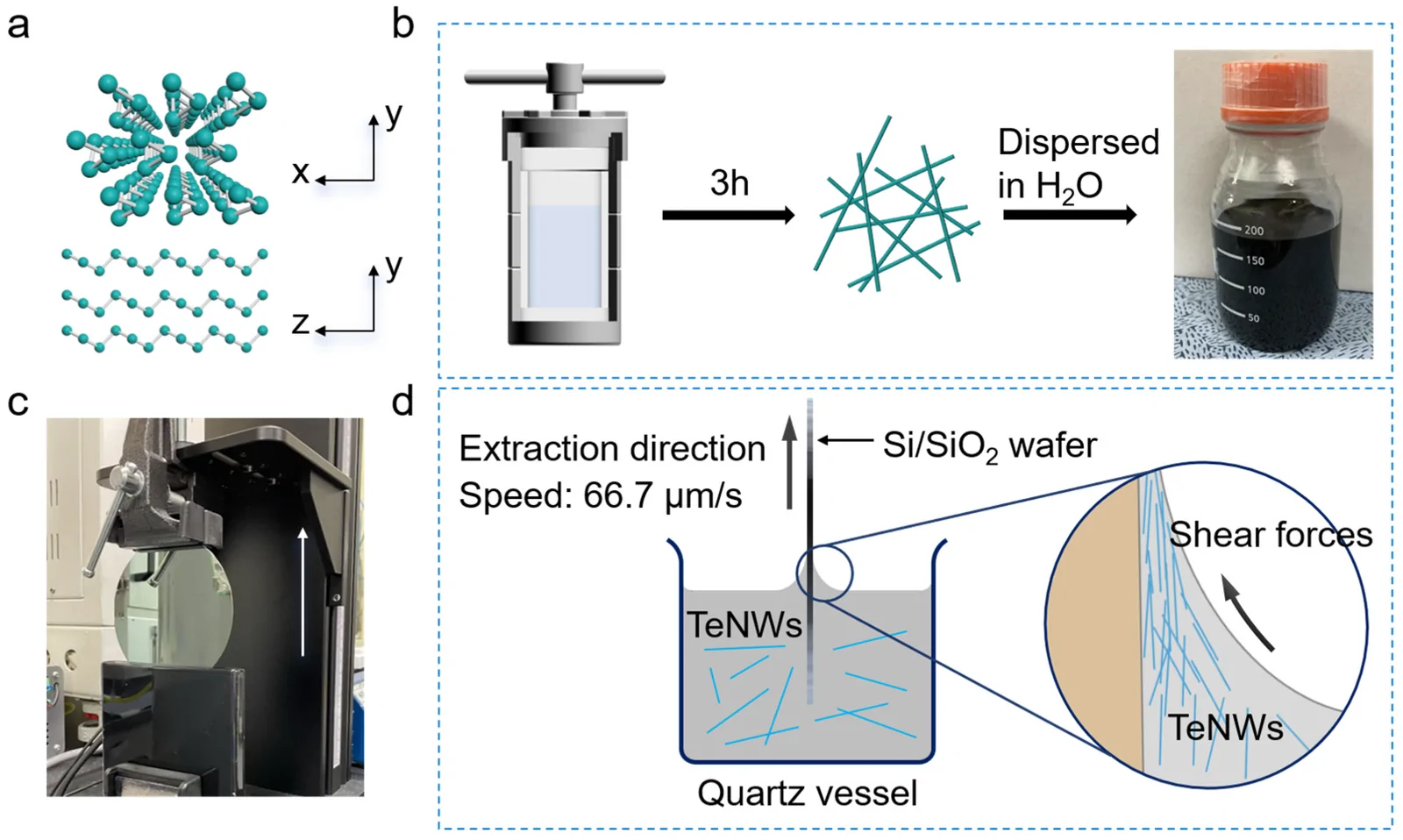
Preparation of TeNWs and solution-shearing self-assembly method. (**a**) Schematic crystal structure of the Te; (**b**) schematic of the synthesis of TeNWs by a hydrothermal method with two steps, including hydrothermal reaction and redispersion; (**c**) photograph of the solution-shearing self-assembly process; (**d**) schematic illustration of the solution-shearing self-assembly mechanism.

Figure 1a presents a schematic diagram of the Te crystal structure, featuring triangular helical chains through the covalent bonding of adjacent Te atoms. These chains extend along the z-axis and are stacked into hexagonal crystal arrays, held together by weaker van der Waals interactions [13]. Figure 1b illustrates the conventional hydrothermal method employed for the synthesis of high-quality, ultrathin TeNWs [35]. Following the synthesis, the precipitates were centrifuged twice to remove the large particles and subsequently redispersed into 100 mL of water to obtain the final TeNW dispersion. The detailed process is described in the experimental section. The solution-shearing self-assembly process began by rapidly immersing the wafer substrate into the dispersion, followed by controlled extraction at a constant velocity of 66.7 μm s^−1^. Figure 1c displays a photograph of this assembly setup. The interfacial interactions were further evaluated through measuring contact angles (Figure S1), which reveal wetting behavior between the dispersion and substrate. The contact angle of the TeNW dispersion droplets recorded at 52.4° is lower than that of the H_2_O droplet at 71.45°. This enhanced wettability comes from the surfactant effect of residual PVP molecules adsorbed on the nanowire surface, which reduces liquid-vapor interfacial tension during shearing process. Although the insulating nature of residual PVP contributes to the nanowire junction resistance, it provides an essential steric barrier to confirm long-term dispersion stability and promote smooth liquid meniscus shearing. The solution-shearing self-assembly mechanism is schematically shown in Figure 1d [37,39]. Shear forces arise from the relative motion of a fluid against a solid surface or another fluid. In other words, shear forces are generated during processes such as surface wetting and de-wetting or when two surfaces move in opposing directions. Here, during the relative movement between the substrate and dispersion fluid, the dispersion is subjected to shear forces from the substrate. TeNWs suspended in the liquid change direction under the influence of the shear force and adhere to the substrate surface. In proper viscosity and shear speed, the TeNWs will align parallel to the direction of the shear force [40,41,42].

**Figure 2:**
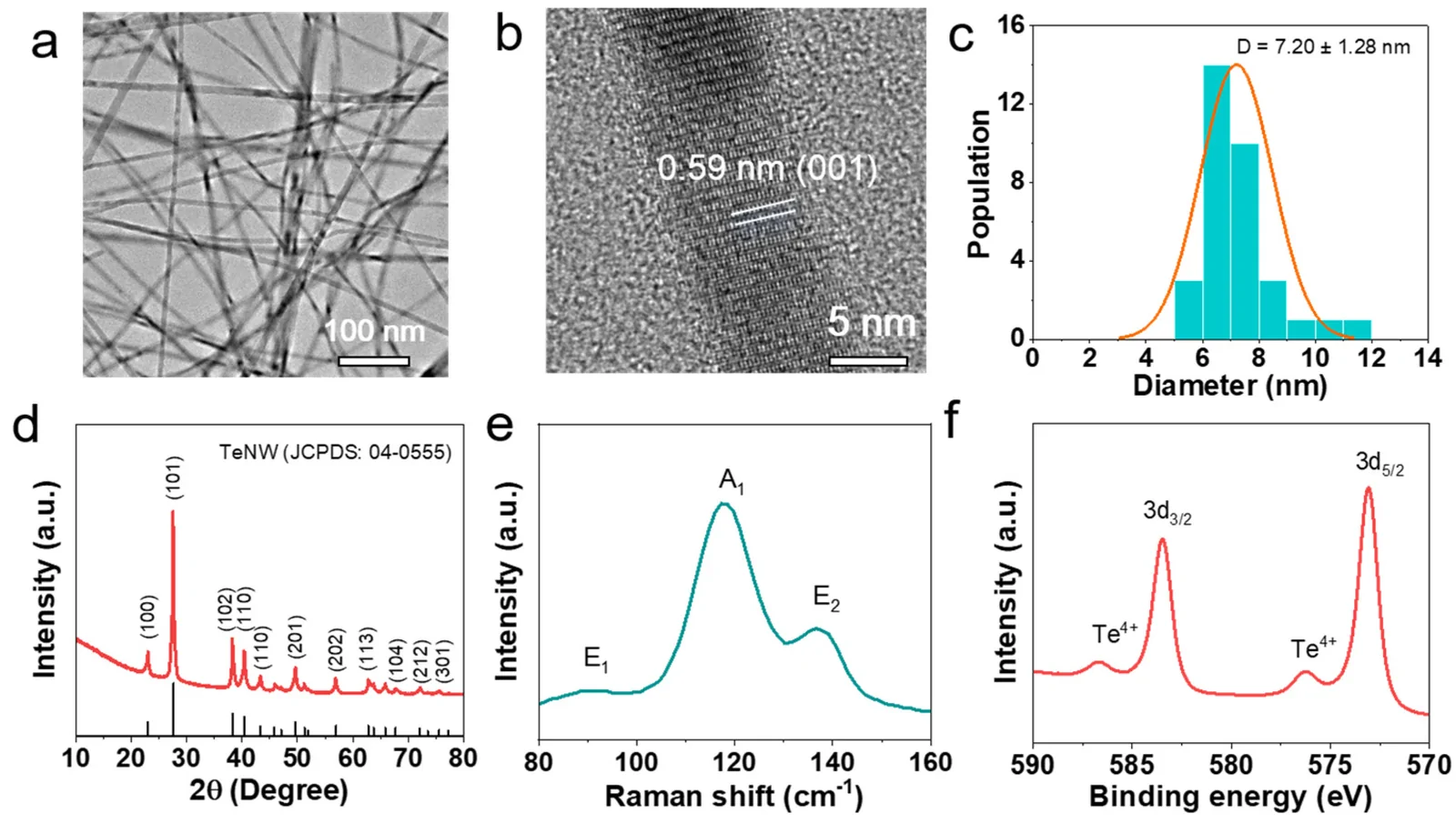
Structural and compositional characterization of the as-synthesized TeNWs. (**a**) Low- magnification bright-field and (**b**) high-resolution electron micrograph of TeNWs; (**c**) Statistical diameter distribution of TeNWs; (**d**) XRD pattern of the TeNWs; (**e**) Raman spectrum of the TeNWs; (**f**) Te 3d XPS spectra of the TeNWs.

Figure 2 shows the structural and compositional characterization of the as-synthesized TeNWs. The morphology of the TeNWs was confirmed using TEM. The bright-field electron micrograph shown in Figure 2a shows the 1-dimensional nanowire morphology with uniform diameters and micrometer-scale length, and the high-resolution electron micrograph shown in Figure 2b confirms the well-defined crystalline structure. The clear lattice fringe spacing of about 0.59 nm indicates that the nanowire was grown along the [001] axis direction. Using Fiji software (ImageJ 1.54p, National Institutes of Health, Bethesda, MD, USA), the diameter is statistically calculated to be 7.2 ± 1.28 nm, and the statistical graph is shown in Figure 2c. The structure and phase of the TeNW powder were examined using XRD, as shown in Figure 2d. The XRD pattern reveals a trigonal crystalline structure of the Te, and all the diffraction peaks are consistent with the trigonal phase of Te (JCPDS, no. 04-0555), indicating the high-crystalline and high-purity nature of the synthesized TeNWs.

To further determine the structure of TeNWs, the Raman spectroscopy is characterized, and the result is shown in Figure 2e. The characteristic peaks at 88.9 cm^−1^, 117.3 cm^−1^, and 136.6 cm^−1^ were observed at room temperature, corresponding to the three main vibration modes: E_1_ (bond-bending), A_1_ (chain expansion), and E_2_ (bond stretching), respectively [43,44]. Notice that the Raman peak wavenumbers are shifted to lower energies compared to those of the previous reports. This can be explained by a weakening of intrachain bonds (covalent bonds) and strengthening interchain interactions (van der Waals forces) [24,45,46,47]. In addition, the chemical composition of the TeNWs was analyzed using X-ray photoelectron spectroscopy (XPS), as shown in Figure 2f. The Te 3d peaks at 583.5 and 573.1 eV correspond to the 3d_3/2_ and 3d_5/2_ spin-orbital doublets of the Te-Te bond, respectively. The Te^4+^ peaks at 586.7 and 576.2 eV are associated with the Te-O bond, which can be attributed to the oxidation by air exposure under ambient conditions [48].

**Figure 3:**
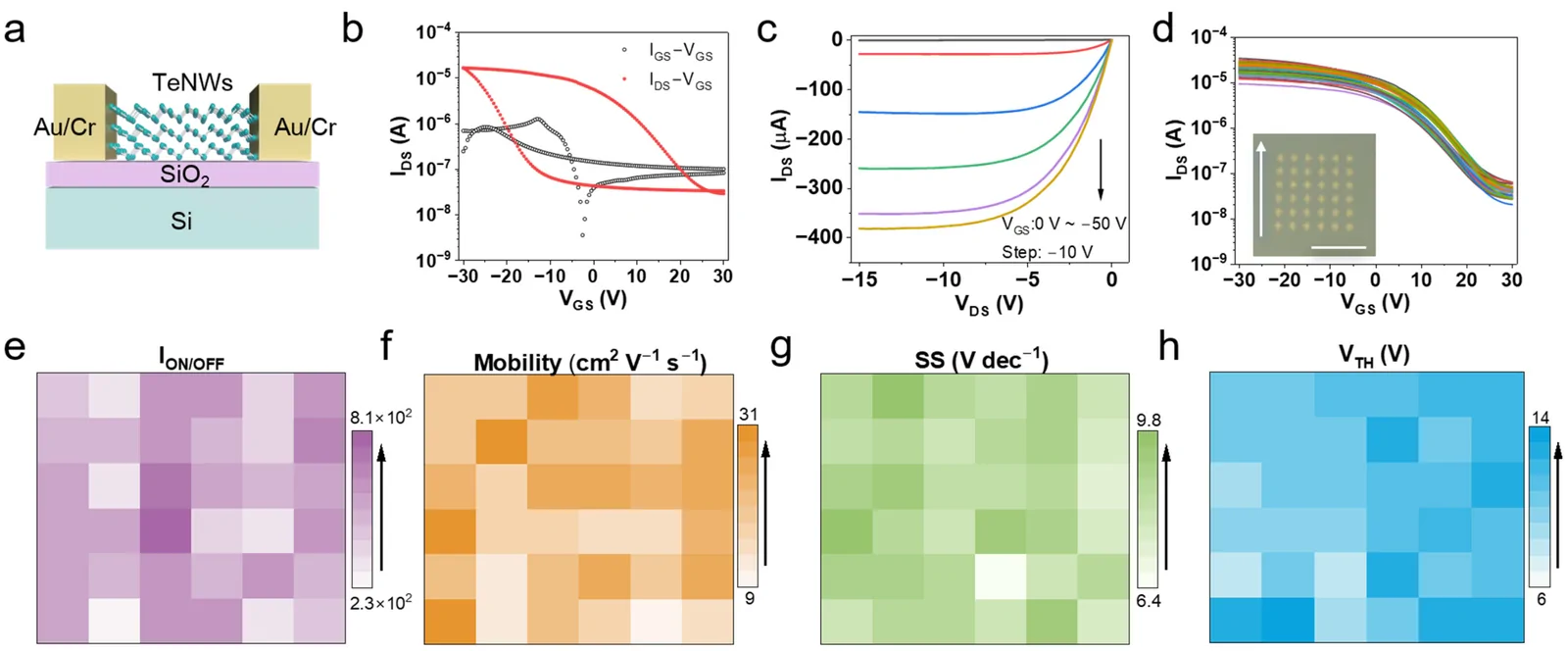
Schematic diagram and electrical characteristics of the fabricated TeNW-FETs. (**a**) Schematic structure of the TeNW-FET; (**b**) transfer curve (I_DS_, red dots) and gate leakage current (I_GS_, black circles) of the TeNW-FET measured at V_DS_ = −0.1 V; (**c**) output curve of TeNW-FET at different V_GS_ ranging from 0 to −50 V at an interval of −10 V; (**d**) transfer characteristics of the 6 × 6 FET array devices. Inset: Optical micrograph of 6 × 6 FET array. Bar scale: 10 mm; (**e**–**h**) statistical distribution mapping of the performance of 36 devices in terms of the (**e**) I_ON/OFF_, (**f**) μ_eff_, (**g**) subthreshold swing (SS) and (**h**) threshold voltage (V_TH_).

Figure 3 exhibits the schematic diagram and electrical characteristics of the fabricated TeNW-FETs. The schematic structure of the TeNW-FET is depicted in Figure 3a. A 5 nm Cr layer was deposited to serve as an adhesion promotor between electrode and nanowire network [49]. Following the device fabrication, the electrical performance was further characterized and depicted in Figure 3b. The transfer characteristic of TeNW-FET (red curve) shows clear p-type behavior with an I_ON/OFF_ ratio of 4.63 × 10^2^ at a V_DS_ of −0.1 V. simultaneously, the gate leakage current (I_GS_, black curve) was recorded to evaluate dielectric insulation within the operational gate voltage regime; the I_GS_ remains consistently much lower than I_DS_, confirming that the modulation of the drain current arises from field-effect carrier accumulation in the TeNW channel rather than from parasitic gate conduction. The linear field-effect mobility of the devices was extracted in the linear operating regime using the following equation:

$$\mu_{\mathrm{lin}}=\frac{L}{WC_{\mathrm{OX}}V_{\mathrm{DS}}}g_{m}=\frac{L}{WC_{\mathrm{OX}}V_{\mathrm{DS}}}\left(\frac{\partial I_{\mathrm{DS}}}{\partial V_{\mathrm{GS}}}\right)$$

where L is the channel length, W is the channel width, C_OX_ is the gate-dielectric capacitance, and g_m_ is the maximum transconductance extracted from the transfer curve at V_DS_ = −0.1 V. Because it is lower than the gate overdrive voltage |V_GS_ − V_TH_| ≈ 15–40 V, the linear-regime approximation condition |V_DS_| << |V_GS_ − V_TH_| is strictly satisfied. The effective field-effect mobility μ_eff_ is the linear field-effect mobility extracted at V_DS_ = −0.1 V. Under these conditions, the μ_eff_ of the representative device in Figure 3b is determined to be 19.7 cm^2^ V^−1^ s^−1^. Figure 3c shows the output characteristics of the TeNW-FET, recorded at V_DS_ ranging from 0 V to −15 V and V_GS_ varied between 0 V and −50 V. The device exhibits typical transistor behavior, characterized by a linear region at low |V_DS_| and a saturation region at high |V_DS_|, confirming that the I_DS_ is effectively modulated by the V_GS_.

To evaluate the uniformity of the TeNWs on the substrate, the electrical performance was measured using a fabricated 6 × 6 FET array containing 36 FETs, as shown in Figure 3d. All 36 FETs exhibit p-type behavior, demonstrating 100% device yield and high uniformity. The inset in Figure 3d shows an optical micrograph of the 6 × 6 FET array, where the arrow denotes the direction of substrate movement during the TeNW self-assembly process. To further analyze the features of the FET array, we examined the electrical characteristics, including I_ON/OFF_, μ_eff_, SS and V_TH_. The statistical mapping of the performance is shown in Figure 3e–h. The 6 × 6 FET devices exhibits the I_ON/OFF_ ratio of (5.1 ± 1.2) × 10^2^, μ_eff_ of 20.6 ± 5.2 cm^2^ V^−1^ s^−1^, V_TH_ of 10.9 ± 1.3 V and SS of 8.4 ± 0.5 V dec^−1^. These results demonstrate that the fabricated FET array exhibits highly uniform transistor characteristics, proving the potential of the solution-shearing strategy for fabricating large-area electronic devices.

**Figure 4:**
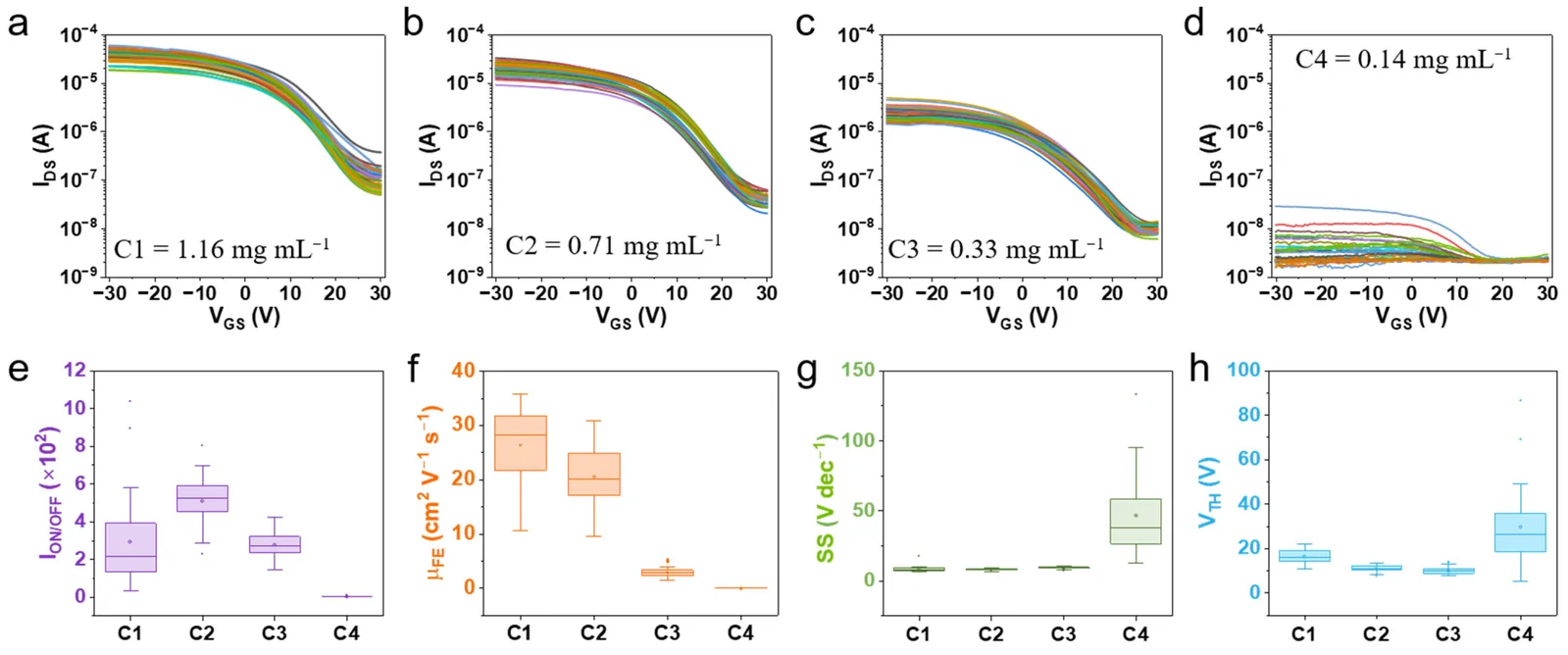
Concentration dependence of electrical performance of TeNW-FETs. (**a**–**d**) The transfer characteristics of 36 devices of TeNW dispersions at different concentrations of 1.16 mg mL^−1^, 0.71 mg mL^−1^, 0.33 mg mL^−1^, and 0.14 mg mL^−1^; (**e**–**h**) Statistical distribution of the I_ON/OFF_, μ_eff_, SS and V_TH_ of 36 devices. Error bars represent the standard deviation of the measured parameters.

To demonstrate the generality of the solution-shearing strategy, we prepared four TeNW dispersions in different concentrations at volumes of 50 mL, 100 mL, 200 mL, and 400 mL. The concentrations were defined as C1, C2, C3, and C4, respectively. After vigorous agitation to obtain well-dispersed solutions, the concentrations were quantitatively analyzed using UV-vis absorption spectroscopy [38], with detailed parameters provided in the Supplementary Materials (Figure S2). The estimated concentrations were approximately 1.16 mg mL^−1^ at C1, 0.71 mg mL^−1^ at C2, 0.33 mg mL^−1^ at C3, and 0.14 mg mL^−1^ at C4. Then, the 6 × 6 FET arrays were fabricated in the same processing to evaluate the concentration-dependent electrical performance, and the transfer characteristics are shown in Figure 4a–d. It is observed that the electrical performance scales with dispersion concentration, with I_DS_ decreasing gradually as the concentration decreases. Furthermore, the SEM analysis was performed on the TeNWs in the channel, revealing that the density of the TeNW network gradually decreased from C1 to C4; The SEM images are shown in Figure S3.

While the transfer curves of the devices maintain excellent uniformity at concentrations of C1, C2 and C3, further concentration dilution to C4 results in a significantly weakened performance and a near loss of uniformity, with a substantial portion of the devices failing to exhibit any transfer modulation. These observations clearly illustrate that lower concentrations result in insufficient TeNW density, making it challenging to form a continuous conductive percolation network and thereby reducing FET performance and device yield. A comparison of the on-state current (I_ON_) and off-state current (I_OFF_) for these four conditions (Figure S4) showed a simultaneous decrease as the concentration scales down from C1 to C4. Furthermore, the electrical performances were evaluated and compared in terms of I_ON/OFF_, μ_eff_, SS and V_TH_, as presented in Figure 4e–h. From C1 to C3, the average I_ON/OFF_ ratio reaches a maximum value at C2; the lower value at C1 is due to the higher leakage current, which results in a larger I_OFF_ and a lower I_ON/OFF_ ratio. The average μ_eff_ decreased from (26.4 ± 7.0) to (2.9 ± 0.8) cm^2^ V^−1^ s^−1^, the average V_TH_ decreased from 16.4 to 10.0 V, and the average SS increased in a low range from 8.4 to 9.4 V dec^−1^. Subsequently, the detailed parameters of the TeNW-FETs are presented in Table 1.

**Figure 5:**
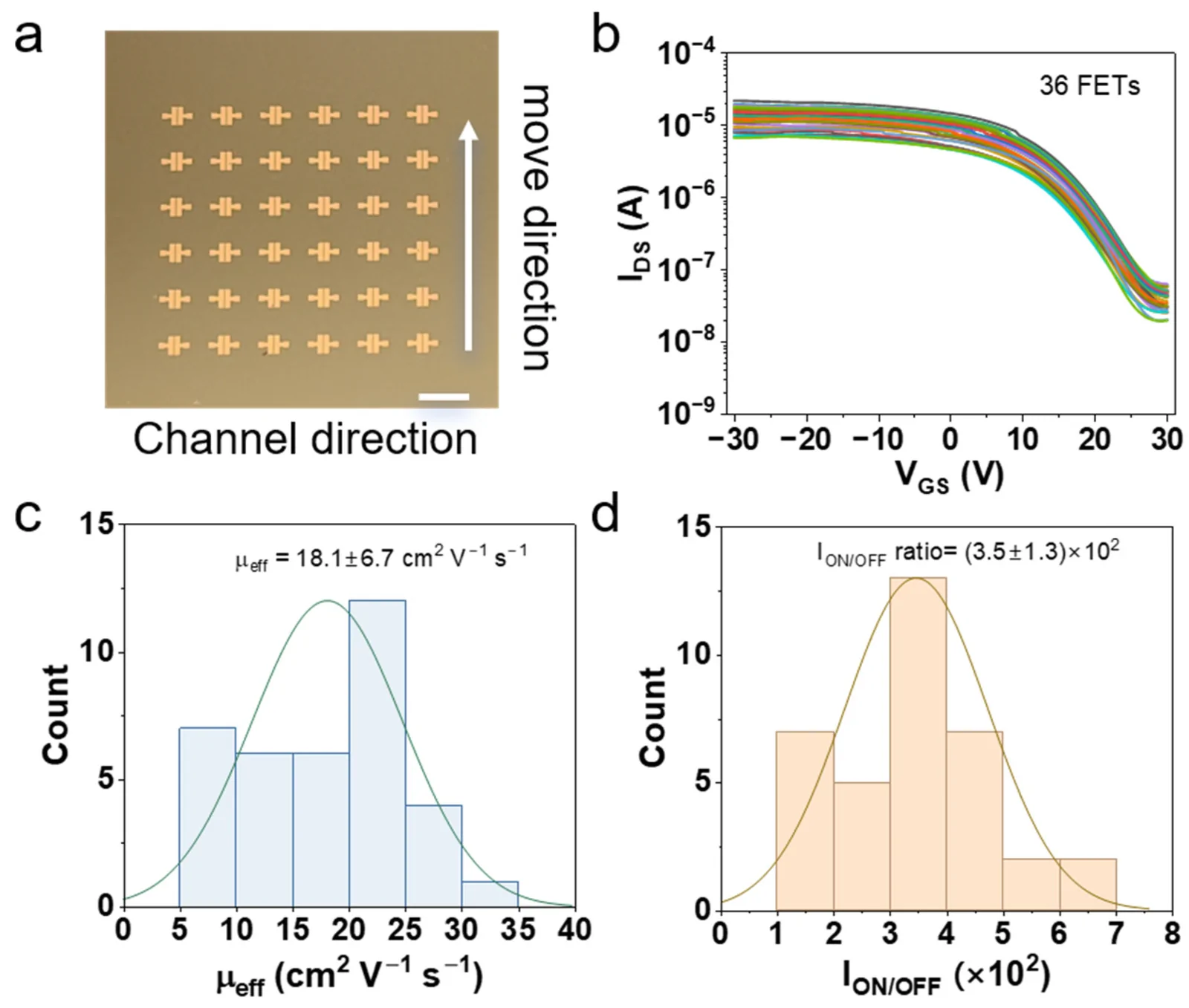
(**a**) The photograph of the fabricated 6 × 6 FET array (white vertical arrow indicates the substrate movement direction during assembly, and white horizontal bar denotes the channel direction; scale bar: 2 mm); (**b**) transfer characteristics of all 36 FETs measured at V_DS_ = −0.1 V; (**c**,**d**) statistical distribution and corresponding fitting curves (solid line) of (**c**) the effective mobility μ_eff_ and (**d**) current on/off ratio (I_ON/OFF_), respectively.

Since the substrate moves in a single direction during the self-assembly process, the directional consistency of the performance is further investigated. We fabricated a 6 × 6 FET array under C2 conditions, with the channel perpendicular to the substrate movement direction; the photograph is shown in Figure 5a. In Figure 5b, the transfer characteristics indicate that all FETs have p-type behavior and high uniformity. The average μ_eff_ and I_ON/OFF_ were statistically calculated as (18.1 ± 6.7) cm^2^ V^−1^ s^−1^ and (3.5 ± 1.3) × 10^2^, respectively, as shown in Figure 5c,d. Compared to the parallel-aligned devices, these values represent (87.9 ± 6.6)% of mobility and (68.6 ± 5.0)% of the I_ON/OFF_ ratio, respectively, reflecting that the mean mobility was nominally reduced by (12.1 ± 6.9)%. This directional performance dependence is mainly due to the percolation transport mechanism in nanowire networks. In such networks, carrier transport is predominantly limited by nanowire−nanowire junction resistances. When the channel current is parallel to the shearing direction, carriers transport across fewer junctions along the channel. In contrast, aligning the channel perpendicular to the shearing direction causes carriers across a higher density of resistive of nanowire−nanowire junctions, resulting in the observed 12.1% mobility reduction and the decrease in on-state current. The statistical graph in Figure S5 shows the preferential orientation of TeNWs along with the substrate movement direction. These results demonstrate higher electrical performance when the channel is aligned parallel to the direction of substrate movement, which may be due to the directional alignment of TeNWs in the channel [38].

**Figure 6:**
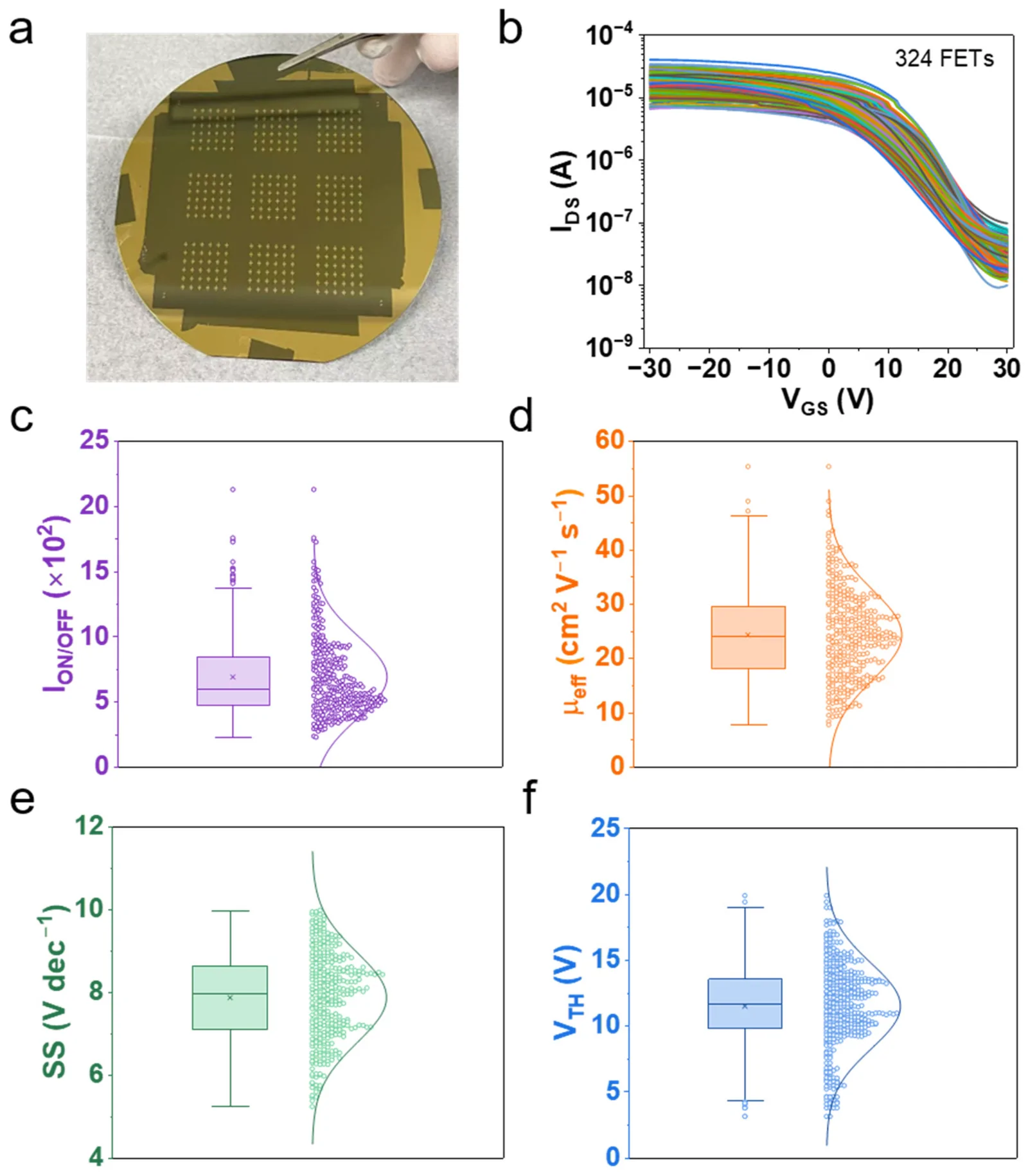
Wafer-scale fabrication and electrical performance distributions of 324 TeNW-FETs. (**a**) Photograph of the TeNW-FET arrays on a 4-inch Si/SiO_2_ wafer; (**b**) transfer characteristics of all 324 devices, showing p-type field-effect operation; (**c**–**f**) distributions of the (**c**) I_ON/OFF_, (**d**) μ_eff_, (**e**) SS and (**f**) V_TH_. Each panel contains a box plot, individual data points, and a density distribution to illustrate the central tendency, spread, and distribution shape of the measured electrical parameters.

To assess the scalability, we fabricated FET arrays on a 4-inch Si/SiO_2_ wafer. Figure 6a shows a photograph of FET arrays containing 324 individual FETs. The transfer characteristics of these devices were measured, and all 324 measured devices exhibited typical p-type behavior without open or short circuits (Figure 6b), corresponding to an observed functional device yield of 100% on the tested wafer. Here, device yield refers to the fraction of devices exhibiting p-type field-effect operation under the measurement conditions used. Figure 6f presents the distributions of the I_ON/OFF_ ratio, μ_eff_, SS and V_TH_, respectively. The corresponding average values were (6.9 ± 3.1) × 10^2^, 24.3 ± 8.2 cm^2^ V^−1^ s^−1^, 7.9 ± 1.1 V dec^−1^ and 12.0 ± 3.0 V. The maximum mobility and I_ON/OFF_ ratio were reached at 55.3 cm^2^ V^−1^ s^−1^ and 2.1 × 10^3^, respectively. Although most devices were concentrated around the central regions of the distributions, the mobility and on/off current ratio exhibited upper tails with notably high values. Despite the distributions revealing some device-to-device variability, all measured devices were fully operational. The variation mainly originated from the local fluctuations in nanowire network density and orientation during the shearing process, which may change the percolation transport path and contact resistances with electrodes. To further illustrate the distribution trends, the 2D schematic distribution mapping of 324 FETs is depicted in Figure S6. The devices exhibit good operational uniformity and a 100% functional device yield. Notably, a subtle spatial gradient is observed along the vertical extraction direction; devices located at the bottom show lower effective mobility and I_ON/OFF_ ratios compared to those in the middle and upper positions. This may be due to the limited stability of TeNW dispersions; gradual sedimentation of TeNWs during the slow shearing process reduces the effective TeNW concentration, causing variation in the nanowire percolation network across the 4-inch wafer. Additionally, nanowire−electrode contact resistance and ambient conditions further contribute to the spread in electrical characteristics. These results demonstrated the feasibility of the solution-shearing process for fabricating transistor arrays on a 4-inch wafer, which provides a promising strategy for the large-scale fabrication of electronic devices.

Employing the solution-shearing self-assembly strategy, we fabricated TeNW-FETs on a 4-inch Si/SiO_2_ wafer with a 100% functional yield across 324 devices and an average μ_eff_ of 24.3 cm^2^ V^−1^ s^−1^. Compared with previously reported low-temperature processing techniques, this approach combines high carrier transport with full 4-inch wafer-scale fabrication without complex substrate pretreatment. This demonstrates that solution-shearing self-assembly is a promising strategy for large-scale, uniform-performing FET fabrication. Nevertheless, it should be noted that several technical challenges remain for mass manufacturing. For instance, the long self-assembling time can lead to a significant portion of the TeNWs settling down in the container, hindering large-scale production. Also, the easy oxidation of TeNWs in the air environment poses a challenge to maintaining device performance. Meanwhile, the immersion-based nature of the process results in extraneous deposition on the wafer edges and backside, necessitating additional post-processing cleaning steps to ensure reliable device characterization. Further process development to address these issues would enable this method to be a viable method for fabricating large-scale electronic devices.

## 4. Conclusions

We employed a solution-shearing self-assembly method to fabricate wafer-scale TeNW-FET arrays. The TeNW dispersion was synthesized via a hydrothermal method and redispersed in DI water. By assembling TeNWs at varying concentrations from 1.16 to 0.14 mg mL^−1^, we demonstrated that the electrical performance of the proposed FETs was strongly influenced by TeNW concentration. Moreover, FETs with channels perpendicular to the substrate movement direction exhibited an average mobility that was approximately (12.1 ± 6.9)% lower than those with channels parallel to the direction, confirming that the fluid shear forces aligned along the movement direction. Finally, large-scale fabrication of FET arrays containing 324 individual devices on a 4-inch Si/SiO_2_ wafer achieved 100% functional device yield and good uniformity. The results show an average I_ON/OFF_ ratio of (6.9 ± 3.1) × 10^2^, a μ_eff_ of 24.3 ± 8.2 cm^2^ V^−1^ s^−1^, an SS of 7.9 ± 1.1 V dec^−1^ and a V_TH_ of 12.0 ± 3.0 V, and a maximum μ_eff_ of 55.3 cm^2^ V^−1^ s^−1^ and I_ON/OFF_ ratio of 2.1 × 10^3^. These findings have significant potential for solution-process manufacturing of large-scale electronic devices.

## Figures and Tables

**Table 1:** Electrical parameters of the TeNW-FETs fabricated by varying concentrations of C1, C2, C3, and C4.

	I_ON/OFF_ Ratio	μ_eff_ (cm^2^ V^−1^ s^−1^)	SS (V dec^−1^)	V_TH_ (V)
C1	(2.9 ± 2.2) × 10^2^	26.4 ± 7.0	8.4 ± 2.0	16.4 ± 3.1
C2	(5.1 ± 1.2) × 10^2^	20.6 ± 5.2	8.4 ± 0.5	10.9 ± 1.3
C3	(2.8 ± 0.7) × 10^2^	2.9 ± 0.8	9.4 ± 0.7	10.0 ± 1.5
C4	20 ± 20	0.004 ± 0.006	46.7 ± 26.6	29.7 ± 18.5

## Data Availability

The original contributions presented in this study are included in the article/Supplementary Material. Further inquiries can be directed to the corresponding authors.

## Supplementary Materials

The following supporting information can be downloaded at: https://www.mdpi.com/article/10.3390/nano16191251/s1, Figure S1: Contact-angle measurements of 1 μL TeNW dispersion droplets (a) and H_2_O (b) on the Si/SiO_2_ wafer substrate; Figure S2: UV-vis absorption spectrum of different TeNW dispersions with 1/10 of their original concentration; Figure S3: SEM images of TeNWs of the devices fabricated by varying dispersion concentrations of 1.16 mg mL^−1^ (a), 0.71 mg mL^−1^ (b), 0.33 mg mL^−1^ (c), and 0.14 mg mL^−1^ (d); Figure S4: Comparison of the on-state current (I_ON_) and off-state current (I_OFF_) as the dispersion concentration reduced from C1 to C4; Figure S5: The statistical graph of TeNW directional alignment of the device channel perpendicular to the substrate movement direction. The 0 degree of deviation means the TeNW direction is parallel to the substrate movement direction. It was observed that about 3% of TeNWs were aligned parallel to the substrate movement direction; Figure S6. Schematic distribution mapping of electrical performance of 324 devices across the 4-inch wafer in terms of (a) I_ON/OFF_, (b) μ_eff_, (c) SS and (d) V_TH_.
