# Supplementary material for: Solution-Shearing Self-Assembly of Hydrothermally Synthesized Tellurium Nanowires for Fabricating Field-Effect Transistors with Wafer-Scale Uniformity
Source: Nanomaterials (Basel). 2026 Oct 3;16(19):1251. doi: 10.3390/nano16191251 (PMC13667251; doi:10.3390/nano16191251)
Supplement: Supplementary file 1 [file nanomaterials-16-01251-s001.zip › nanomaterials-4530936-supplementary.pdf]

Supplementary Information

Result and discussion supplements

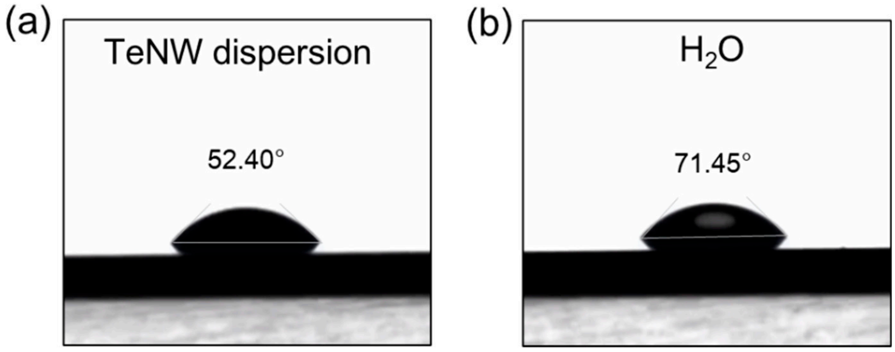

Figure S1. Contact-angle measurements of 1μL TeNW dispersion droplets (a) and H_2O (b) on the Si/SiO_2 wafer substrate.

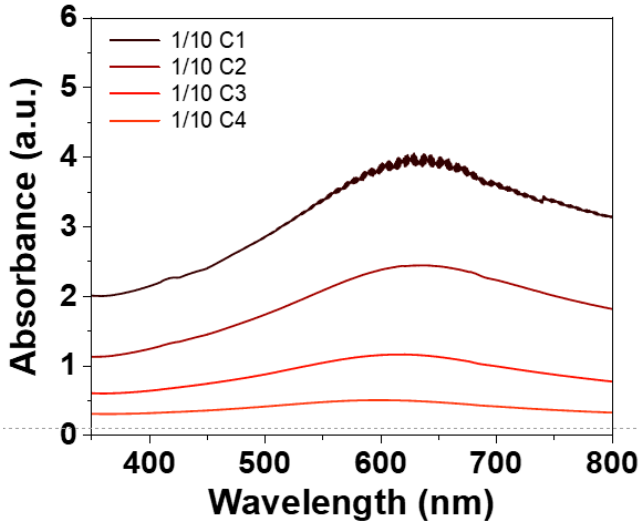

Figure S2. UV-vis absorption spectrum of different TeNW dispersions with 1/10 of their original concentration.

The concentrations of TeNW dispersions were estimated through UV-vis spectroscopy using a literature-based calibration. Each dispersion was diluted 10-fold with deionized water before measurement in a 1 cm path-length cuvette. The absorbance at 659 nm was used to estimate concentration according to the Beer-Lambert equation.

Zhao et al. [1] reported a TeNW dispersion diluted 1500-fold from 19.7 mg mL^{-1}, with an absorbance of approximately 0.448 at 659 nm. The measured absorbances of diluted dispersions C1-C4 were 3.9662, 2.4131, 1.1120 and 0.4709, respectively, giving original concentrations of 1.16 mg mL^{-1}, 0.71 mg mL^{-1}, 0.33 mg mL^{-1} and 0.14 mg mL^{-1}. These values represent approximate concentrations estimated based on a specific reference dilution factor and reference sample.

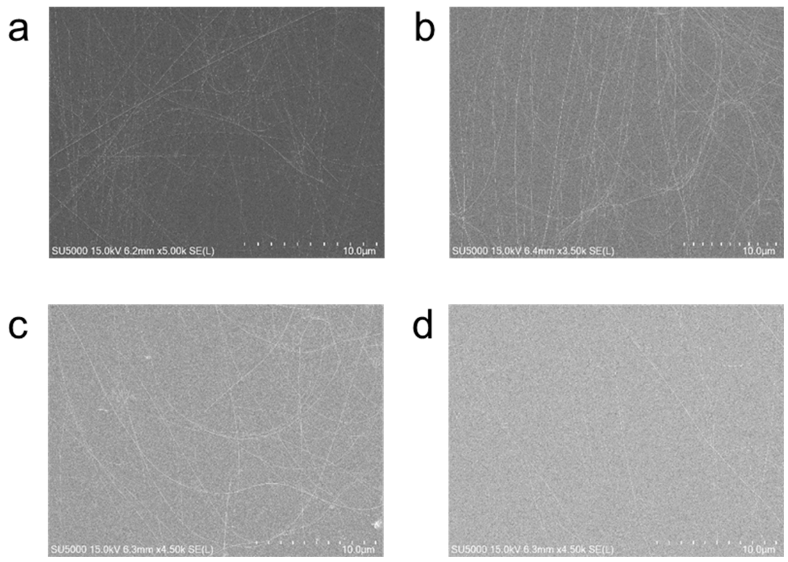

Figure S3. SEM images of TeNWs of the devices fabricated by varying dispersion concentrations of 1.16 mg mL^{-1} (a), 0.71 mg mL^{-1} (b), 0.33 mg mL^{-1} (c), and 0.14 mg mL^{-1} (d).

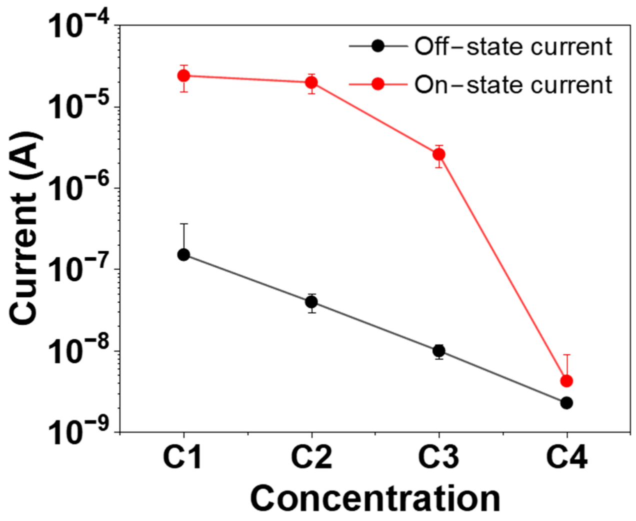

Figure S4. Comparison of the on−state current (I_{ON}) and off−state current (I_{OFF}) as the dispersion concentration reduced from C1 to C4.

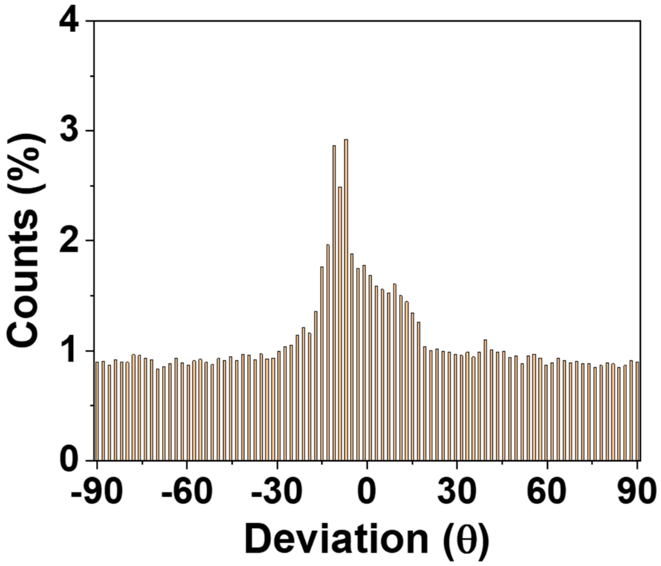

Figure S5. The statistical graph of TeNW directional alignment of the device channel perpendicular to the substrate movement direction. The 0 degree of deviation means the TeNWs direction is parallel to the substrate movement direction. It was observed that about 3% of TeNWs were aligned parallel to the substrate movement direction.

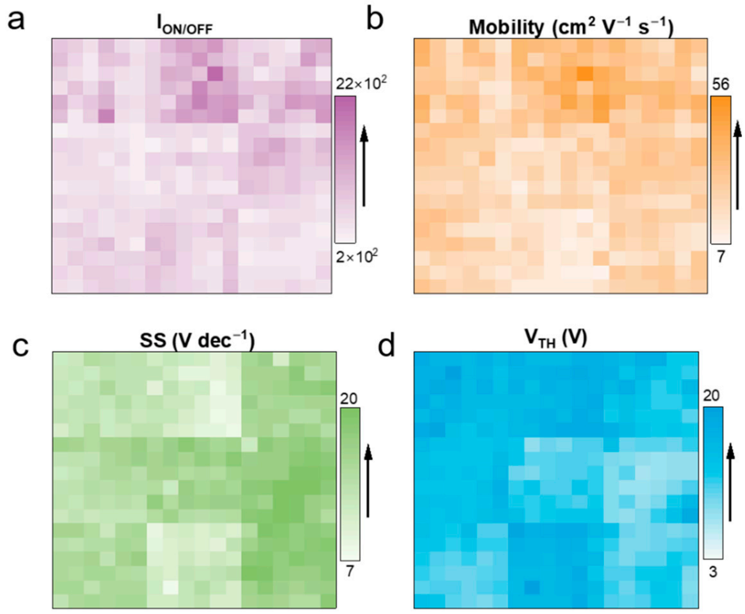

Figure S6. Schematic distribution mapping of electrical performance of 324 devices across the 4-inch wafer in terms of (a) $I_{ON/OFF}$, (b) μ_{eff}, (c) SS and (d) V_{TH}.

References

1. Zhao, Y.; Sanchuan, Z.; Xixi, P.; Anni, Z.; Chenning, L.; Yuxuan, L.; Xiaomeng, D.; Lei, C.; Zhenhua, Y.; Tailang, H.; et al. Biomimetic wafer-scale alignment of tellurium nanowires for high-mobility flexible and stretchable electronics. *Science Advances* **2024**, *10*, 14.
